# Detection of elevated levels of PINK1 in plasma from patients with idiopathic Parkinson’s disease

**DOI:** 10.3389/fnagi.2024.1369014

**Published:** 2024-04-22

**Authors:** Xianchai Hong, Yi Zheng, Jialong Hou, Tao Jiang, Yao Lu, Wenwen Wang, Shuoting Zhou, Qianqian Ye, Chenglong Xie, Jia Li

**Affiliations:** ^1^Department of Neurology Nursing Unit 362 Ward, The First Affiliated Hospital of Wenzhou Medical University, Wenzhou, China; ^2^Department of Neurology, The First Affiliated Hospital of Wenzhou Medical University, Wenzhou, China; ^3^Department of Neurology, Yuhuan City People's Hospital, Taizhou, China; ^4^The Center of Traditional Chinese Medicine, The Second Affiliated Hospital and Yuying Children's Hospital of Wenzhou Medical University, Wenzhou, China; ^5^Key Laboratory of Alzheimer's Disease of Zhejiang Province, Wenzhou City, China; ^6^Institute of Aging, Wenzhou Medical University, Wenzhou, Zhejiang, China; ^7^Oujiang Laboratory, Wenzhou, Zhejiang, China

**Keywords:** PINK1, **α**-synuclein oligomer, biomarkers, mitophagy, Parkinson’s disease

## Abstract

**Backgrounds:**

Numerous lines of evidence support the intricate interplay between Parkinson’s disease (PD) and the PINK1-dependent mitophagy process. This study aimed to evaluate differences in plasma PINK1 levels among idiopathic PD, PD syndromes (PDs), and healthy controls.

**Methods:**

A total of 354 participants were included, consisting of 197 PD patients, 50 PDs patients, and 107 healthy controls were divided into two cohorts, namely the modeling cohort (cohort 1) and the validated cohort (cohort 2). An enzyme-linked immunosorbent assay (ELISA)-based analysis was performed on PINK1 and α-synuclein oligomer (Asy-no). The utilization of the area under the curve (AUC) within the receiver-operating characteristic (ROC) curves served as a robust and comprehensive approach to evaluate and quantify the predictive efficacy of plasma biomarkers alone, as well as combined models, in distinguishing PD patients from controls.

**Results:**

PINK1 and Asy-no were elevated in the plasma of PD and PDs patients compared to healthy controls. The AUCs of PINK1 (0.771) and Asy-no (0.787) were supposed to be potentially eligible plasma biomarkers differentiating PD from controls but could not differentiate PD from PDs. Notably, the PINK + Asy-no + Clinical RBD model showed the highest performance in the modeling cohort and was comparable with the PINK1 + Clinical RBD in the validation cohort. Moreover, there is no significant correlation between PINK1 and UPDRS, MMSE, HAMD, HAMA, RBDQ-HK, and ADL scores.

**Conclusion:**

These findings suggest that elevated PINK1 in plasma holds the potential to serve as a non-invasive tool for distinguishing PD patients from controls. Moreover, the outcomes of our investigation lend support to the plausibility of implementing a feasible blood test in future clinical translation.

## Introduction

Parkinson’s disease (PD) is a prevalent (estimated to occur in about 1% of individuals older than 60 years), chronic neurodegenerative disorder afflicting the elderly and is caused by a together of genetic and environmental factors (Poewe et al., 2017). At present, the clinical diagnosis of idiopathic PD, primarily conducted by movement disorders specialists, relies heavily on the traditionally defined features of bradykinesia, rigidity, and resting tremor, along with non-motor symptoms (NMS). However, this approach suffers from a significant error rate, as evidenced by inconsistencies found in approximately 30–40% of patients clinically diagnosed with PD upon post-mortem examination of their brains (Armstrong and Okun, 2020). Thus, it is critically needed that an accurate and reliable diagnosis strategy be implemented to improve diagnosis and enable disease identification and clinical trial design. To date, numerous molecular biological processes underlying PD pathophysiology have been identified, including mitochondrial dysfunction, protein aggregation, DNA repair damage, and neuroinflammation (Armstrong and Okun, 2020). There is substantial evidence supporting an association between PD and mitophagy, which removes damaged or superfluous mitochondria (Pickrell and Youle, 2015; Ryan et al., 2015). Hence, an easily accessible biomarker that could reflect the status of mitophagy function possibly as an objective measure for diagnosing PD and improving the prediction of disease progression.

PINK1, a serine/threonine protein kinase primarily localized within mitochondria, has emerged as a key modulator of mitophagy (Villa et al., 2018). Mutations in PINK1 have been linked to autosomal recessive PD, with affected patients manifesting impaired mitochondrial quality control mechanisms (Valente et al., 2004; Ivatt et al., 2014). Mitophagy, a selective process involving the autophagic removal of excess or damaged mitochondria, serves as a critical process in maintaining mitochondrial homeostasis in eukaryotic cells (Lou et al., 2019). Notably, studies in mammalian cells have demonstrated that Parkin, a cytosolic protein, is recruited to dysfunctional mitochondria in a PINK1-dependent manner, facilitating their engulfment and subsequent degradation (Whitworth and Pallanck, 2017). Recent studies have revealed that PINK1–Parkin-mediated mitophagy is the main molecular mechanism pathway in this process (Palikaras et al., 2018).

There is a growing consensus on the potential diagnostic and prognostic utility of Cerebrospinal Fluid (CSF) and blood biomarkers in PD, offering insights into the underlying pathophysiology (Parnetti et al., 2019). Blood-based biomarkers are preferable to those in the CSF or other fluids as blood can be easily collected (Ashton et al., 2020). However, PINK1 is not well studied as a biomarker of mitophagy status in PD, with limited investigations into plasma mitophagy function or PINK1 expression in terms of PD subjects (Hsieh et al., 2016). Hence, the primary objective of this pilot study was to evaluate the performance of plasma PINK1 levels as a diagnostic biomarker for idiopathic PD and as a predictor of disease severity.

## Methods

### Standard protocol approvals, registrations, and patient consent

All patients included in this study were consecutively enrolled from the First Affiliated Hospital of Wenzhou Medical University, a prominent primary medical unit located in Wenzhou, between March 2018 and May 2022. Participants recruited after March 2020 were designated as Cohort 1 (Modeling cohort) while the remaining individuals were assigned to Cohort 2 (Validation cohort). Ethical approval for this study was obtained from the institutional Ethics Board Committee of the Wenzhou Medical University First Affiliated Hospital, and written informed consent was obtained from all participants prior to their involvement in the research. The primary objective of our investigation was to ascertain the potential of plasma PINK1 levels as an underlying diagnostic biomarker for idiopathic PD diagnosis, as well as to differentiate patients with PD syndromes [PDs: e.g., Multiple System Atrophy (MSA), Progressive Supranuclear Palsy (PSP), or Vascular Parkinsonism (VPD)] from those with idiopathic PD and analyze the association of PINK1 levels with motor or non-motor performance and other related factors in patients with PD.

### Study populations

**Figure 1:**
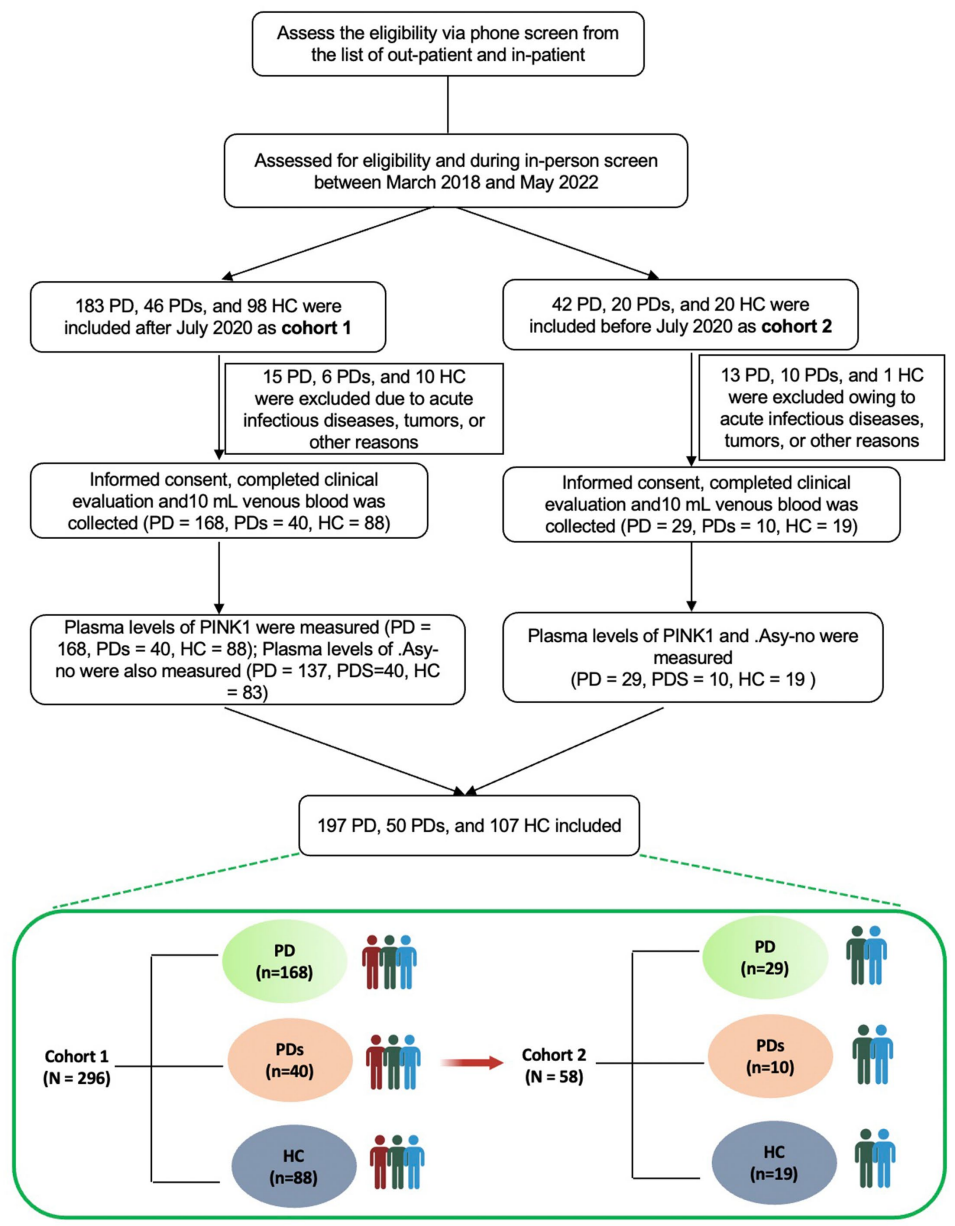
Flow chart of the study design and selection process.

See Figure 1 for a comprehensive flowchart illustrating the selection process utilized in this study. The study cohort consisted of a total of 354 participants, comprising 197 patients diagnosed with PD, 50 patients diagnosed with PDs, and 107 healthy controls (HCs). The diagnosis of PD was established based on the widely accepted Movement Disorder Society (MDS) clinical diagnostic criteria (Postuma et al., 2015). Patients diagnosed with MSA adhered to the consensus statement for the diagnosis of this specific disorder. HCs were recruited from the same institute and were selected among spouses or accompanying friends of patients with PD or PDs, ensuring neurologically normal conditions. Participants with a history of stroke, tumors, acute infectious diseases, and other relevant conditions were excluded from the study. This study was cross-sectional, without a longitudinal follow-up design. All subjects underwent a comprehensive cross-sectional clinical assessment, encompassing demographic and clinical data. Motor severity was evaluated with Unified Parkinson’s Disease Rating Scale (UPDRS) part III motor scores and with Hoehn-Yahr staging (H-Y). Non-motor was assessed via the Mini-Mental State Examination (MMSE), Hamilton Depression Rating Scale (HAMD), Hamilton Anxiety Rating Scale (HAMA), REM sleep behavior disorder questionnaire-Hong Kong (RBDQ-HK), and revised scale of Activity of Daily Living (ADL). Additionally, levodopa equivalent daily dose (LEDD) and PD complications, including falls, constipation, dyskinesia, and on–off phenomena were also assessed.

### Clinical evaluation

Respectively, we evaluated motor symptom severity and progression stage of PD with a modified UPDRS (Elton, 1987) and H-Y staging (Hoehn and Yahr, 1967). Patients with H-Y stage <3 points were classified as early-stage PD and those with stage ≥3 were classified as advanced PD. Cognitive function was evaluated using the Chinese version of the Mini-Mental State Examination (MMSE), with adjustment of the cutoff score for cognitive impairment according to the level of education as follows: illiterate, ≤ 17 points; primary school education, ≤ 20 points; and postsecondary education or above, ≤ 24 points (Katzman et al., 1988; Cui et al., 2011). PD with dementia (PDD) was diagnosed based on the criteria proposed by the Movement Disorder Society (Dubois et al., 2007). The HAMD (defined as possible depression, 7–17; depression, 17–24; and severe depression, >24) and HAMA (defined as possible anxiety, 7–14; anxiety, 14–21; and severe anxiety, >21) scales were performed as reflected emotional conditions. The RBDQ-HK was used to detect RBD, with a cutoff value of >18 points (Li et al., 2010). All assessments were conducted during the “on” phase of the disease.

### Measurement of plasma biomarkers

At the time of enrollment, a total of 10 mL of venous blood was collected from each participant. Within 1–2 h of collection, the blood samples underwent centrifugation at 3,000 *g* for 10 min to separate the plasma. Subsequently, plasma aliquots were carefully transferred into cryotubes and immediately stored at a temperature of −80°C until further analysis. Plasma levels of PINK1 and Asy-no were measured with a PINK1 assay kit (Jianglai Biotechnology Company, Shanghai, China, http://www.jonln.com; No: JL11175) and an Asy-no kit (JL41188) according to the manufacturer’s instructions by research assistants who were blinded to the clinical diagnosis.

### Statistical analysis

The clinical and demographic characteristics of the participants were summarized in Table 1, with continuous variables presented as mean ± SD for those following a normal distribution, and as medians (25th and 75th percentiles) for variables with an abnormal distribution. Categorical variables were expressed as counts and percentages. Normality assessments for the dataset employed a suite of statistical evaluations, including the Kolmogorov–Smirnov test and visual inspections via P–P and Q-Q plots. Analysis of covariance (ANCOVA) or the Kruskal-Wallis test, followed by Bonferroni corrected *post hoc* comparisons, were utilized for continuous variables data analysis. Chi-square tests facilitated categorical variable comparisons, with adjustments made via Bonferroni corrections. Univariate and multivariable logistic regression analyses were conducted with the results reported as adjusted odds ratios (OR) along with their corresponding 95% confidence intervals (CIs). A directed acyclic graph (DAG), derived from Bayesian network principles (Fleischer and Diez Roux, 2008) was used with the “bnlearn” R package to guide the causal relationship among different covariates including demographic characteristics (sex, age, BMI, education, smoker, drinker, hypertension pressure as well as diabetes mellitus), neuropsychological assessments (MMSE, HAMA, HAMD, ADL, UPDRS, H-Y stage, and RBDQ-HK), non-motor status according to neurological assessments (cognitive impairment, depression, anxiety, and RBD), LEDD, and diagnosis.

**Table 1:** Characteristics for the modeling and validated cohorts.

Characteristics	Modeling cohort						Validated cohort					
	HC (*N* = 88)	PD (*N* = 168)	PDs (*N* = 40)	PD vs. HC	HC vs. PDs	PD vs. PDs	HC (*N* = 19)	PD (*N* = 29)	PDs (*N* = 10)	PD vs. HC	PDs vs. HC	PD vs. PDs
Age (years)^a^	64.5 [58.8;69.0]	67.0 [60.8;72.0]	67.5 [62.0;73.5]	0.043	0.043	0.402	68.0 [61.0; 74.0]	63.0 [62.0; 69.0]	75.0 [68.2; 78.0]	0.375	0.027	0.098
Female (%)^b^	49 (55.7%)	74 (44.0%)	17 (42.5%)	0.304	0.350	1.000	13 (68.4%)	17 (58.6%)	4 (40.0%)	0.703	0.697	0.697
Height (cm)^a^	162 (7.02)	161 (8.30)	161 (8.90)	0.693	0.676	0.863	159 (7.03)	160 (8.61)	160 (6.40)	0.849	1.000	0.912
Weight (kg)^a^	63.6 (9.48)	61.8 (10.3)	61.8 (9.96)	0.354	0.343	0.605	62.7 (8.17)	63.1 (17.5)	62.8 (8.75)	0.992	0.998	1.000
BMI^a^	24.3 (2.98)	23.9 (3.26)	24.0 (4.40)	0.643	0.619	0.866	24.7 (2.54)	24.3 (5.02)	24.6 (4.31)	0.947	0.985	0.997
Education (years)^a^	5.00 [0.00;8.00]	4.00 [0.00;7.00]	3.00 [0.00;6.25]	0.835	0.835	0.835	2.00 [0.00; 4.50]	3.00 [0.00; 8.00]	2.50 [0.00; 4.00]	0.576	0.576	0.962
Smoker (%)^b^	13 (14.8%)	37 (22.0%)	12 (30.0%)	0.331	0.228	0.389	1 (5.26%)	3 (10.3%)	2 (20.0%)	1.000	0.881	0.800
Drinker (%)^b^	16 (18.2%)	42 (25.0%)	9 (22.5%)	0.840	0.900	0.900	2 (10.5%)	4 (13.8%)	5 (50.0%)	1.000	0.048	0.048
HP (%)^b^	40 (45.5%)	56 (33.3%)	16 (40.0%)	0.232	0.701	0.701	15 (78.9%)	9 (31.0%)	7 (70.0%)	0.009	0.090	0.665
DM (%)^b^	16 (18.2%)	29 (17.3%)	11 (27.5%)	0.991	0.503	0.503	0 (0.00%)	4 (13.8%)	1 (10.0%)	0.426	1.000	0.517
Disease history (years)^a^	-	3.00 [1.00;7.00]	2.00 [1.00;4.00]	-	-	0.003	-	4.00 [2.00; 5.00]	2.75 [1.25; 6.50]	-	-	<0.001
UPDRS^a^	-	40.0 [27.0;53.0]	49.0 [34.5;67.0]	-	-	0.076	-	36.0 [28.0; 52.0]	62.5 [56.5; 74.2]	-	-	<0.001
I^a^	-	2.00 [1.00;4.00]	3.00 [2.00;5.00]	-	-	0.004	-	2.00 [1.00; 3.00]	3.50 [2.00; 6.25]	-	-	<0.001
II^a^	-	11.0 [7.00;16.0]	13.0 [10.5;18.2]	-	-	0.051	-	11.0 [10.0; 14.0]	17.5 [13.2; 20.0]	-	-	<0.001
III^a^	-	24.0 [15.0;35.0]	30.0 [16.5;39.2]	-	-	0.302	-	23.0 [14.0; 33.0]	39.5 [27.8; 46.8]	-	-	<0.001
IV^a^	-	1.00 [0.00;3.00]	1.00 [0.00;2.25]	-	-	0.420	-	3.00 [0.00; 5.00]	3.50 [1.25; 4.75]	-	-	<0.001
H-Y stage^a^	-	2.50 [1.50;3.00]	3.00 [2.00;4.00]	-	-	0.007	-	2.00 [1.50; 2.50]	3.00 [3.00; 4.00]	-	-	<0.001
MMSE^a^	24.0 [21.0;26.0]	23.0 [18.0;26.0]	16.0 [11.0;20.8]	0.126	<0.001	<0.001	24.0 [20.0; 27.0]	26.0 [20.0; 28.0]	17.5 [11.5; 20.0]	0.899	0.061	0.061
HAMD^a^	3.00 [0.00;5.00]	5.00 [2.75;9.00]	5.00 [4.00;9.00]	<0.001	<0.001	0.440	2.00 [0.00; 5.50]	6.00 [3.00; 9.00]	7.00 [3.50; 8.50]	0.022	0.884	0.034
HAMA^a^	4.00 [1.00;7.00]	8.00 [4.00;13.0]	8.00 [4.00;11.2]	<0.001	<0.001	0.809	3.00 [0.00; 9.00]	14.0 [8.00; 17.0]	7.00 [5.25; 8.75]	<0.001	0.036	0.152
RBDQ-HK^a^	3.00 [1.00;10.0]	13.0 [3.00;31.0]	7.00 [2.75;26.2]	<0.001	0.005	0.327	5.00 [3.50; 7.50]	24.0 [8.00; 46.0]	12.5 [3.75; 38.2]	0.003	0.520	0.273
ADL^a^	20.0 [20.0;20.0]	26.5 [21.0;35.0]	35.5 [26.2;48.2]	<0.001	<0.001	0.001	20.0 [20.0; 20.0]	22.0 [20.0; 26.0]	50.5 [31.2; 58.8]	<0.001	0.001	<0.001
PINK1 (ng/mL)^a^	77.1 (15.9)	96.2 (20.0)	96.4 (20.3)	<0.001	<0.001	0.996	66.6 (4.62)	74.5 (6.72)	71.8 (6.10)	<0.001	0.438	0.076
Asy-no (pg/mL)^ac^	2631 (667)	3352 (645)	3445 (625)	<0.001	<0.001	0.701	2406 (209)	2494 (236)	2574 (209)	0.381	0.595	0.141

Continuous variables were assessed for normality by PP plot and QQ plot. Data are expressed as mean (SD), median [IQR], or *n* (%); ^a^*p* values obtained from One-Way ANOVA or Kruskal-Wallis test followed by Bonferroni corrected *post hoc* comparisons, and UPDRS and H-Y stage were only compared between PD and PDs by Mann–Whitney U test; ^b^*p* values obtained from chi-squared test corrected by Bonferroni. HC, Healthy control; PD, Parkinson disease; PDs, Parkinsonian syndrome; Smoker: people who have smoked continuously or cumulatively for 6 months or more throughout their lives; Drinker: people who have drinked continuously or cumulatively for 6 months or more throughout their lives; HP, Hyper blood pressure; DM, Diabetes mellitus; UPDRS, Unified Parkinson’s disease rating scale; MMSE, Mini-Mental State Examination; HAMD, Hamilton depression scale; HAMA, Hamilton anxiety scale; RBDQ-HK, REM sleep behavior disorder questionnaire-Hong Kong; ADL, Activity of daily living scale; BMI, Body mass index; Asy-no, Oligomeric α-syn; PINK1, PTEN induced putative kinase 1. ^c^Less participants took the examination of plasma a-synuclein oligomer levels in the modeling cohort (HC = 83, PD = 137, and PDs = 40).

Pearson correlation analysis served to elucidate the association between PINK1 and asymptomatic norms (Asy-no). Additionally, the associations between PINK1 levels and neuropsychological evaluation scales were investigated employing restricted cubic spline (RCS) (Desquilbet and Mariotti, 2010), with adjustments for age, sex, and education. The mediation effect was analyzed leveraging the “mediation” R package (Klumparendt et al., 2019). Then, the predictive utility of plasma biomarkers, both in alone or combined models, in differentiating among PD, PDs, and controls was appraised through Receiver Operating Characteristic (ROC) curve analysis, specifically examining the area under the curve (AUC), and the differences in the AUC were determined using DeLong statistics. To compare the difference of plasma PINK1 levels in subgroups, we used two-tailed *t*-tests. The ROC analysis and the combined models were first practiced in cohort 1 and subsequently validated in cohort 2 to assess the stability of the models. Other analyses were practiced in both cohorts. Statistical analyses were conducted using R version 4.1 and SPSS version 26. Statistical significance was defined at a two-tailed *p* < 0.05.

## Results

### Demographics, disease characteristics of the cohorts

Demographics and disease characteristics of the three groups (HCs, PD, and PDs) are presented in Table 1. For the modeling cohort, the groups were matched by age and gender distribution (PD vs. HC or PD vs. PDs), with PD groups were also comparable in terms of other demographic and clinical factors such as female rate, height, weight, BMI, education, smoking and drinking habits, blood pressure, and presence of diabetes mellitus when compared to control groups. On average, participants with PD were 2.5 years older than those in the HC group, and this was similar when compared to the PDs group. Additionally, significant differences were observed in various assessment scales, including the UPDRS, H-Y stage, HAMA, HAMD, RBDQ-HK, ADL scores, and levels of PINK1 and Asy-no, between PD and controls. Furthermore, our findings indicate that patients with PDs exhibited more severe motor symptoms and psychological conditions compared to PD participants, as evidenced by the assessment scales both in the modeling cohort and validated cohort.

### PINK1 is elevated in the plasma of PD patients

**Figure 2:**
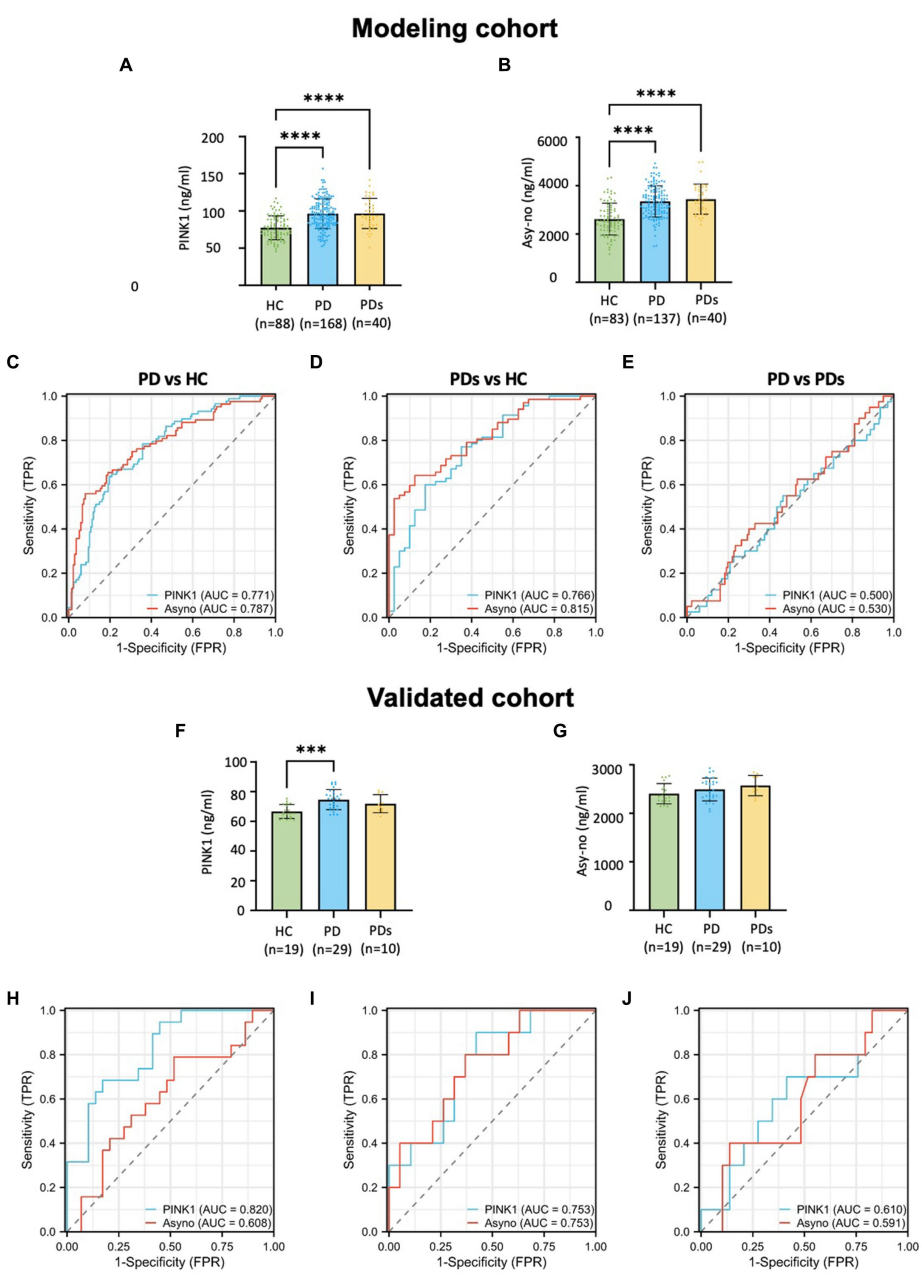
Plasma-derived PINK1 and Asy-no concentrations and diagnostic accuracy across clinically defined diagnostic profiles in modeling and validated cohort. Distribution of PINK1 and Asy-no concentrations across clinically defined diagnostics groups in modeling cohort **(A,B)** and validated cohort **(F,G)**; The diagnosis accuracy of PINK1 and Asy-no in the context of PD vs. HC, PDs vs. HC, and PD vs. PDs in modeling cohort **(C–E)** and validated cohort **(H–J)**. ^*^p < 0.05, ^**^p < 0.01, ^***^p < 0.001, and ^****^p < 0.0001 compared to HC group.

Figure 2 demonstrates the plasma concentration of PINK1 and Asy-no in HCs, PD, and PDs groups. Previously, several genetic studies revealed that the PINK1 gene mutated in autosomal recessive parkinsonism. PINK1 is known to play a pivotal role in mitochondrial quality control, providing further evidence of mitochondrial involvement in PD (Pickrell and Youle, 2015; Clark et al., 2021). Here, we found that plasma PINK1 concentration was significantly higher in both the PD (96.2 ± 20.0 ng/mL) and PDs (96.4 ± 20.3 ng/mL) as compared to the HC group (77.7 ± 15.9 ng/mL). In this cohort, the level of PINK1 was comparable between PD and PDs, in cases and controls (Figure 2A). To assess the utility of plasma PINK1 levels in discriminating between idiopathic PD and HC, we obtained a 256 cross-section of samples from the modeling cohort (Table 1). The ROC curve had an AUC of 0.771 for PINK1 with an optimum threshold was 80.404, and corresponding sensitivity and specificity were, respectively, 63.6 and 80.4% (Figure 2C). Similarly, when comparing PDs patients against the HC group, the AUC for PINK1 was 0.766 (Figure 2D). Notably, the AUC for PINK1 in discriminating between PD and PDs was 0.500 (Figure 2E), indicating that plasma PINK1 levels do not provide discriminatory value in distinguishing PD from PDs.

Moreover, Asy-no, a protein implicated in PD, is considered one of the main pathological factors in the disease. However, the precise mechanisms through which it exerts its neurotoxic effects remain unclear (Lobanova et al., 2022). Recent evidence indicates that the assembly of toxic oligomeric species of a-synuclein may play a crucial role in the pathology and spread of PD (Bengoa-Vergniory et al., 2017). Analysis of samples across the study period revealed a nominal elevation in Asy-no levels in PD (3,352 ± 645 pg./mL) and PDs (3,445 ± 625 pg./mL) compared to controls (2,631 ± 667 pg./mL) in the modeling cohort (Figure 2B). As a classical diagnostic marker, the AUC values for Asy-no were comparable to those of PINK1. The ROC analysis showed that a plasma Asy-no cutoff value of 2595.7 pg./mL exhibited a sensitivity of 56.0% and a specificity of 92.0% for distinguishing between PD and HC, with an AUC of 0.787 (Figure 2C). Furthermore, the Asy-no levels were higher in PDs compared to age-matched controls, with an AUC of 0.815 (Figure 2D). However, the AUC for Asy-no was 0.530 between PD and PDs (Figure 2E). In the validation cohort, PINK1 levels were elevated in PD compared to HC groups, with no discernible differences observed between PD and PDs (Figures 2F,G). There was no significant difference in PINK1 levels between HC and PDs, and Asy-no levels remained unchanged across the three groups, possibly attributed to the small sample size. Moreover, the AUCs of the PINK1 and Asy-no were 0.820 and 0.608, in PD vs. HCs (Figure 2H), 0.753 and 0.753 when PDs vs. HC (Figure 2I), while for PD vs. PDs, they were 0.610 and 0.591 (Figure 2J), respectively. The diagnostic performance of the PINK1 and Asy-no was evaluated by the AUC values in the training cohort and independently verified in the validation cohort.

### Estimation of the performance of combined models and verification

**Figure 3:**
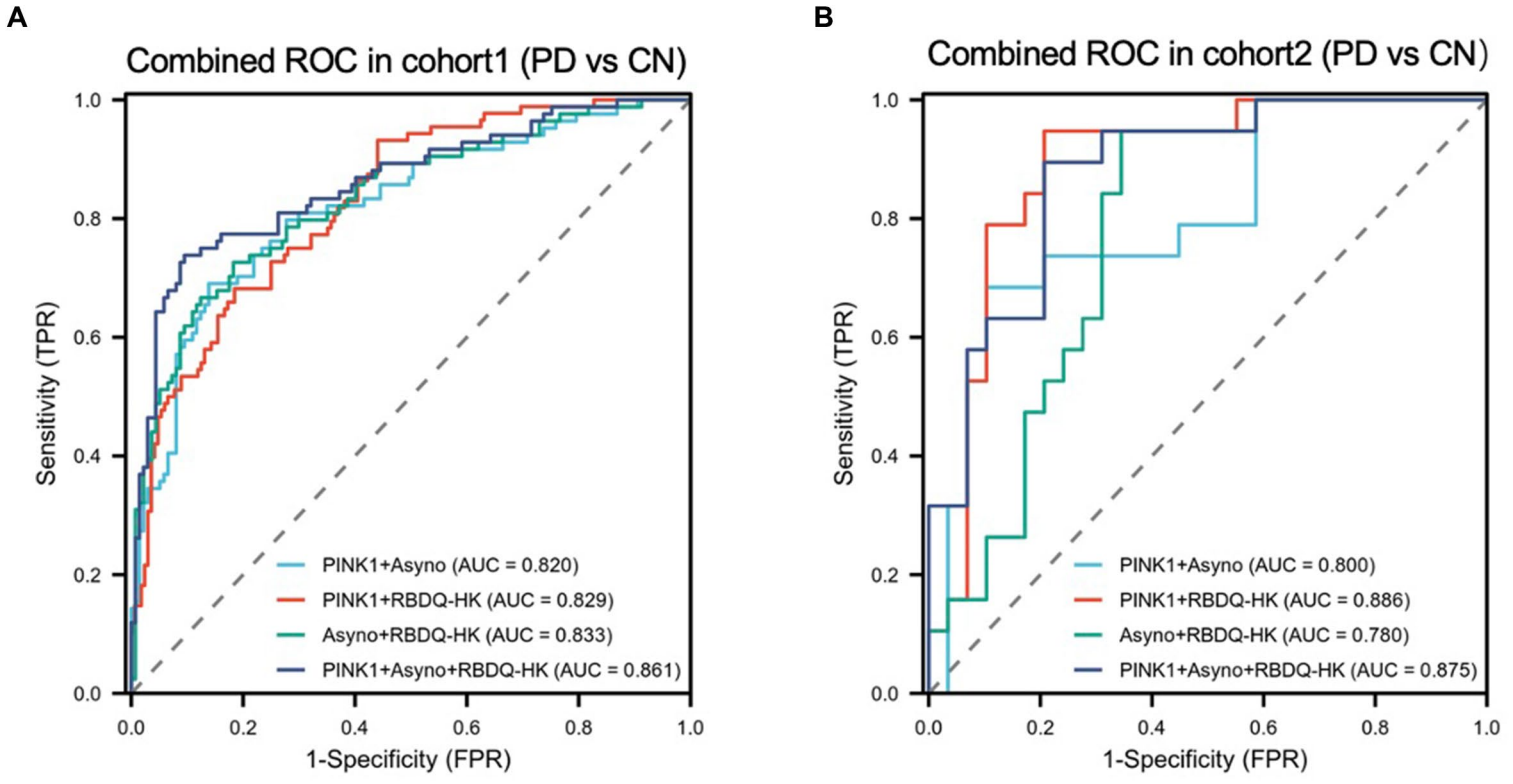
The receiver operating characteristic (ROC) curve analyzes the diagnosis accuracy of combined models to identify PD from HC participants in the modeling cohort and validated cohort. Four combined models (model1: PINK1 + Asyno; model2: PINK1 + RBDQ-HK; model3: Asyno + RBDQ-HK; and model4: PINK1 + Asyno + RBDQ-HK) were constructed by multivariate logical regression to differentiate PD from HC. Receiver operating characteristic (ROC) curve analysis of combined models was used to confirm diagnosis accuracy in the modeling cohort **(A)** and the results were validated in the validated cohort **(B)**.

To determine whether demographic characteristics and neuropsychological scale levels were independently associated with PD. We conducted a two-step analysis consisting of a univariate analysis followed by a multivariable logistic regression (Supplementary Table 1) and found RBDQ-HK score is a suitable predictor of PD diagnosis. In order to develop a more precise and clinically applicable combined model for diagnosing PD, we evaluated various combined models in both the Modeling cohort and the validation cohort. In the Modeling cohort (Figure 3A; Supplementary Table 2), the AUCs of the combined models constructed with PINK1 + Asy-no, PINK1 + Clinical RBD, Asy-no + Clinical RBD, and PINK + Asy-no + Clinical RBD were 0.820 (95%CI 0.761–0.879), 0.829 (95%CI 0.763–0.875), 0.833 (95%CI 0.777–0.890), and 0.861 (95%CI 0.808–0.913). Similarly, in the validation cohort (Figure 3B), the AUCs of the combined models built with PINK1 + Asy-no, PINK1 + Clinical RBD, Asy-no + Clinical RBD, and PINK + Asy-no + Clinical RBD were 0.800, 0.886, 0.780, and 0.875. Among these models, the PINK + Asy-no + Clinical RBD model showed the highest performance in the modeling cohort and exhibited comparable performance to the PINK1 + Clinical RBD in the validation cohort (Figure 3).

### Association of plasma PINK1 with clinical characteristics, motor and nonmotor features

**Figure 4:**
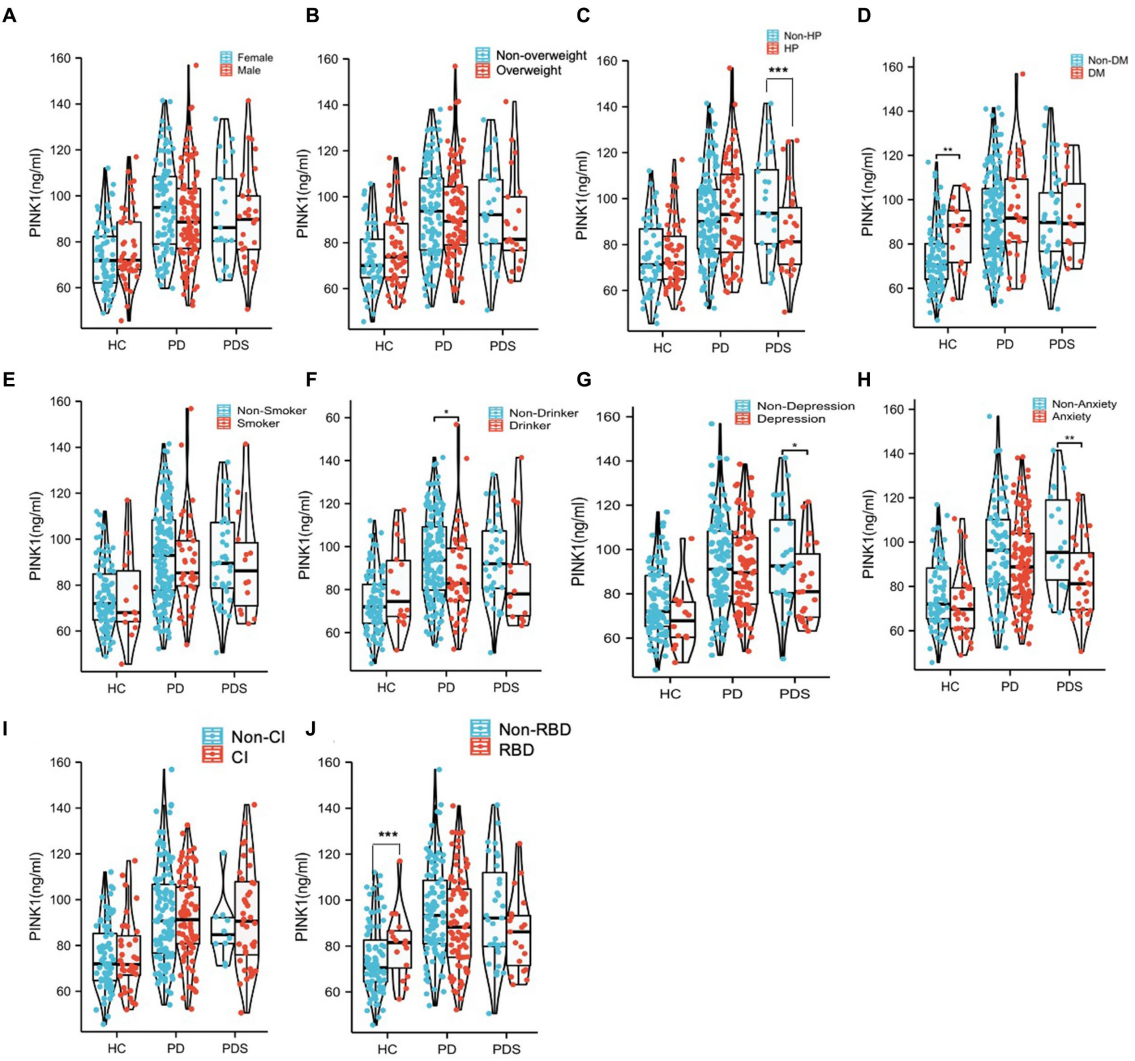
The comparison of PINK1 levels in the 10 subgroups with different clinical diagnoses. All the subjects in different diagnostic groups were redivided into subgroups according to different factors including Sex **(A)**, Overweight **(B)**, HP **(C)**, DM **(D)**, Smoker **(E)**, Drinker **(F)**, Depression **(G)**, Anxiety **(H)**, CI **(I)**, and RBD **(J)**. ^*^p < 0.05, ^**^p < 0.01. Overweight, subjects with BMI ≥ 24; HP, subjects with high blood pressure; DM subjects with diabetes mellitus; Smoker, subjects who have smoked continuously or cumulatively for 6 months or more in their lifetime; Drinker, subjects who have consumed alcohol continuously or cumulatively for 6 months or more in their lifetime (at least once a week); Anxiety, subjects with possible anxiety or anxiety and HAMA score ≥ 7; Depression, subjects with possible depression or depression and HAMD score ≥ 7; and CI, subjects with cognitive impairment according to MMSE scores (MMSE was used for cognitive examination, with adjustment of the cutoff score for cognitive impairment according to the years of education as follows: illiterate, ≤17 points; primary school education, ≤20 points; and postsecondary education or above, ≤24 points); RBD, subjects with RBDQ-HK >18 points.

**Figure 5:**
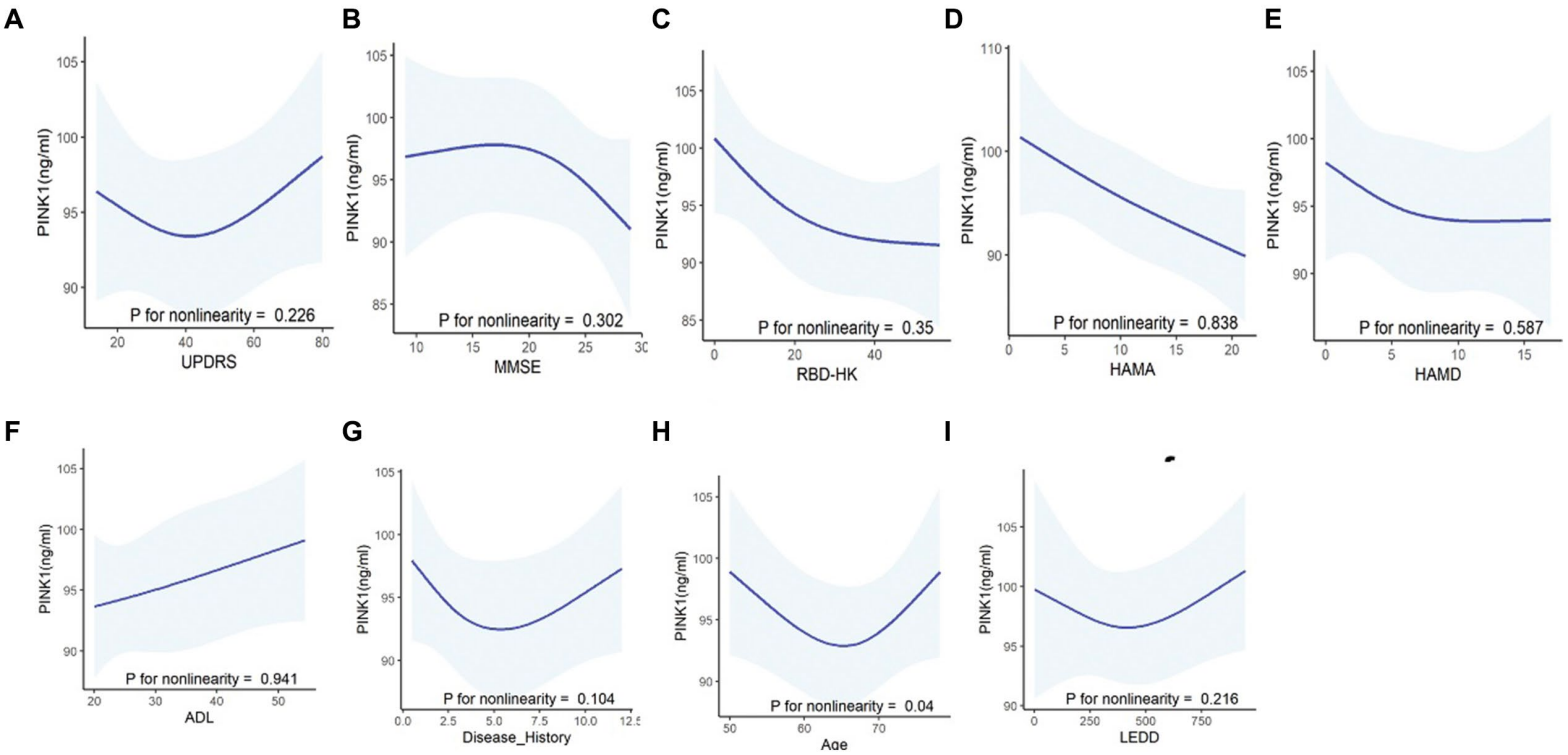
Correlations between PINK1 levels and clinical assessments in PD patients using the restricted cubic spline curves (RCS) fitting model. Using non-linear smoothing spline regressions RCS model between PINK1 as a continuous value and neuropsychological evaluation scales including UPDRS **(A)**, MMSE **(B)**, RBDQ-HK **(C)**, HAMA **(D)**, HAMD **(E)**, and ADL **(F)** and other clinical values such as disease history **(G)** and LEDD **(I)** after adjusting age, sex, and education. The correlation between PINK1 level and age **(H)** was assessed after adjusting sex and education. *p* > 0.05, indicating lack of correlation of the tested values.

To evaluate the potential impact of demographic characteristics and neuropsychological assessment scores on PINK1 concentration, our study conducted a comprehensive analysis. The results showed there were no significant differences in PINK1 levels between male and female participants (Figure 4A), overweight status (Chinese BMI classification; Pan et al., 2021, underweight was defined as <18.5, normal weight as 18.5–24, overweight as 24–28, and obesity as ≥28, Figure 4B), smoker or not (Figure 4E), as well as cognition impairment in the three groups (Figure 4I). Moreover, the levels of PINK1 were significantly lower in the PDs patients with hypertension (Figure 4C), depression (Figure 4G), and anxiety (Figure 4H). Conversely, in the control group, elevated PINK1 expression was associated with diabetes mellitus (Figure 4D) and subjects with RBD (Figure 4J). Subsequently, PD patients were stratified based on alcohol consumption, revealing higher PINK1 levels in the group of drinkers (Figure 4F). The detailed data can be found in the Supplementary Table 3. Additionally, employing restricted cubic spline (RCS) models allowed for a nuanced exploration of the relationship between plasma PINK1 levels, treated as a continuous variable, and neuropsychological evaluation scales, while adjusting for age and sex. Interestingly, our analysis revealed no significant positive association between plasma PINK1 levels and UPDRS (*p* = 0.226), MMSE (*p* = 0.302), RBD-HK (*p* = 0.35), HAMA (*p* = 0.838), HAMD (*p* = 0.587), ADL (*p* = 0.941), disease duration (*p* = 0.104), and LEDD (*p* = 0.216) in PD subjects, except age with a U-shape association (*p* = 0.04; Figure 5).

### PINK1 mediates the association between Asy-no and the risk of PD incident

**Figure 6:**
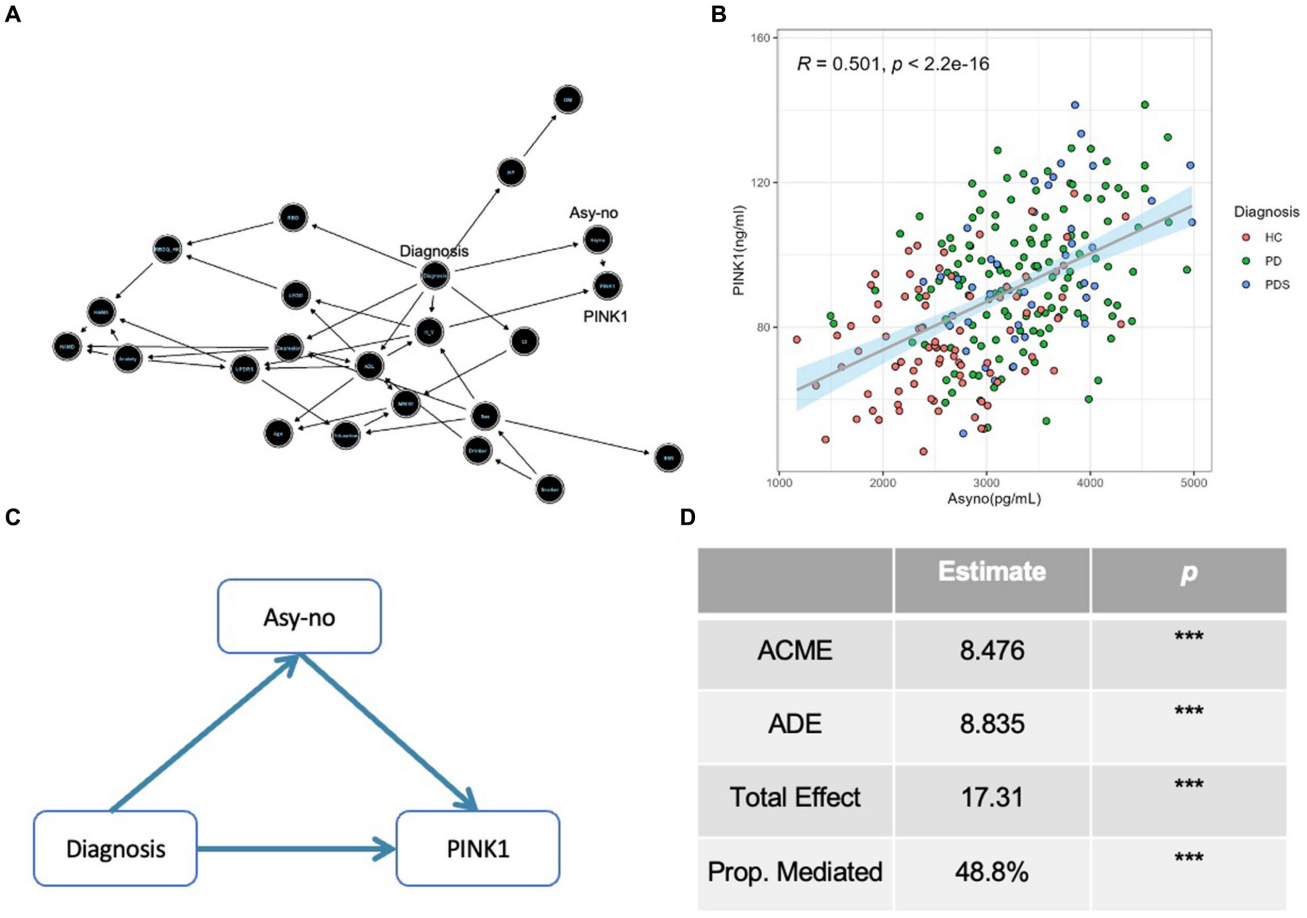
The Diagnosis-Asyno-PINK1 pathway was validated in different analyses. **(A)** A directed acyclic graph (DAG) based on the Bayesian network was used to assess the causal relationship among different covariates including demographic characteristics (sex, age, BMI, education, smoker, drinker, hypertension pressure as well as diabetes mellitus), neuropsychological assessments (MMSE, HAMA, HAMD, ADL, UPDRS, H-Y stage, and RBDQ-HK), non-motor status according to neuropsychological assessments (cognitive impairment, depression, anxiety, and RBD), LEDD, and diagnosis. **(B)** A curve of linear regression was used to show the relationship between PINK1 and Asy-no levels. The correlation coefficient (*r*) and *p* value were assessed by Pearson correlation. **(C)** A diagram of mediation effect analysis was used to show the Diagnosis-Asyno-PINK1 pathway and the proportion of mediation effect is 48.8% **(D)**. ACME, Average causal mediation effect; ADE, Average direct effect; Prop. Mediated, proportion mediated; ^*^p < 0.05, ^**^p < 0.01, and ^***^p < 0.001 in mediation effect analysis.

The DAG was used to guide the causal relationship among different covariates, which is a graphical tool that visually represents and enhances our comprehension of exposure, outcome, causation, and bias (Williams et al., 2018). As seen in Figure 6A, an arrow from PD incident diagnosis to Asy-no signifies our hypothesis that changes in Asy-no levels may influence the occurrence of PD. Moreover, the arrow from Asy-no to PINK1 indicates the close relationship between these two factors in the PD diagnosis domain. Further, our study revealed a significant correlation between plasma Asy-no and PINK1 concentrations, as demonstrated by a linear correlation analysis model (correlation coefficient *r* = 0.501, *p* < 0.001, Figure 6B). This observation suggests a potential influence of Asy-no development on PINK1 levels. Notably, mediation analysis was performed for the Asy-no and PINK1, revealing that the relationship between Asy-no and the risk of PD incidence was significantly mediated by the PINK1 element (Proportion of mediation = 48.8%, Figures 6C,D). Due to this analysis representing an assumption, we should tone down the claim that PINK1 mediates the relationship between Asy-no and the risk of PD when treating these results.

## Discussion

The challenge of accurately diagnosing Parkinson’s Disease (PD) is compounded by the broad spectrum of symptomatology observed across individuals and the absence of definitive diagnostic tools. In terms of the therapy domain, despite numerous tested molecules, there is still a lack of agents or neuroprotective therapies capable of effectively slowing down, halting, or reversing neurodegeneration in PD patients, partly due to the administration being applied too late. Therefore, there is a pressing need to identify easily measurable and highly predictive biomarkers for early-stage PD (Parnetti et al., 2019). Genetic studies in PD highlight the *pink1* gene in disease risk (Valente et al., 2004; Gegg and Schapira, 2011). However, the role of *pink1* in disease progression remains inadequately elucidated. To our knowledge, no previous study has biochemically assessed PINK1 activity markers in plasma from PD cases.

Presently, we found that (1) the PINK1 and Asy-no are elevated in the plasma of PD and PDs patients compared to healthy controls. (2) The AUCs of PINK1 and Asy-no were considered as potential eligible plasma risk factors differentiating PD from controls but could not differentiate PD from PDs. They showed similar discriminatory power between the PD and control groups, with AUCs ranging between 0.771 and 0.787. (3) There is no significant correlation between PINK1 and UPDRS, MMSE, HAMD, HAMA, RBDQ-HK, and ADL scores. Although the PINK1 level does not appear to relate to the neuropsychological tests, we found that individuals with PD develop higher PINK1 levels. These findings provide insights into the potential use of plasma PINK1 as a latent diagnostic biomarker, comparable to Asy-no, and support the future implementation of a feasible blood test in clinical practice. However, at present, such data are not enough to justify the routine suggestion that PINK1 can be supposed as a PD diagnostic biomarker in the clinic. Specifically, although levels seem to be elevated in PD as compared to healthy control in both the modeling and validation cohorts, there is apparent overlap in data points and not adequate effect size differentiating PD from controls. Accordingly, the reported sensitivity for PINK1 levels in the modeling cohort is 64%, which is low for a proposed Biomarker. Therefore, we should be cautious when explaining these results.

The dysregulation of the mitophagy-related protein PINK1, as reported in our study, offers valuable clues to the pathogenesis of PD. Recently, a series of research have revealed the mechanisms by which two enzymes, PINK1 and Parkin, mediate a mitochondrial quality control system, which contributes to the removal of damaged mitochondria through the mitophagy process (McWilliams and Muqit, 2017). Biochemical studies conducted in transfected cells and transgenic mice have provided evidence that damaged mitochondria activate the mitochondrial-associated kinase PINK1, leading to the phosphorylation of both ubiquitin and Parkin at their respective Ser65 locus (Pickrell and Youle, 2015; Lou et al., 2019). This triggers a feedforward amplification cascade of mitochondrial ubiquitylation, ultimately resulting in the clearance of the damaged organelles through mitophagy (Ordureau et al., 2014). Moreover, as impaired mitophagy has been connected to early-onset PD results from the mutations in human PINK1 and Parkin genes, aberrant mitophagy emerged as a promising hypothesis to illustrate the underlying pathophysiology of this disorder (Whitworth and Pallanck, 2017). Despite PINK1 being recognized as a key regulator of mitophagy and its involvement in PD pathogenesis, little is known about its exact activity in the plasma of PD subjects. Currently, in this project, using the ELISA-based approaches, we found that the PINK1 is elevated in the plasma of PD patients compared to healthy controls. The AUC of PINK1 is considered as a potential eligible plasma biomarker differentiating PD from controls. These results are consistent with the theory that mitochondrial dysfunction in PD patients would be associated with higher mitophagy-related protein levels as a reflection of toxic mitophagy induction. Furthermore, the potential of PINK1 as a diagnostic biomarker was strengthened in two independent cohorts (AUC 0.771 and 0.820), even though the sample size is relatively small in the second cohort. Notably, PINK1 is not well studied in blood biofluid as a biomarker of mitophagy status in humans. While we found a high level of PINK1 in the plasma of PD and PDs patients compared to healthy controls, these data cannot be directly correlated with mitophagy pathway induction at present. Anyway, these findings lay the groundwork for future investigations into other biomarkers related to the mitophagy pathway in plasma or other biofluids to reflect neurodegeneration in PD. More biochemical investigation and clinical exploration might be required to understand the role.

Interestingly, our study has demonstrated that the combined assessment of PINK1 and Asy-no profiles could potentially serve as a robust composite biomarker model for PD in human samples, as indicated by a notable AUC value of 0.820. It is noteworthy that the PINK1-dependent mitophagy process closely interacts with a-synuclein aggregation in this disease. In PD models, the accumulation of a-synuclein leads to an upregulation of Miro protein levels on the surface of mitochondria, resulting in a delay in the mitophagy process (Shaltouki et al., 2018). However, it has been shown that the reduction of Miro levels can rescue mitophagy phenotypes and alleviate neurodegeneration in neurons derived from PD subjects. Moreover, the regulatory circuit formed by PINK1, Parkin, and a-synuclein plays a crucial role in modulating the mitochondrial stress response, providing a potential physiological basis for the prevalence of a-synuclein pathology in PD (Norris et al., 2015). A transgenic mouse strain with specific overexpression of the A53T mutant form of a-synuclein in dopamine neurons exhibits profound early-onset mitochondrial abnormalities, further emphasizing the critical involvement of mitochondria and the consequential defective mitophagy in the pathogenesis of PD (Chen et al., 2015). Indeed, α-synuclein and PINK1 are two critical proteins associated with the pathogenesis of PD. Their interaction stimulated the removal of excess α-synuclein, which prevented mitochondrial deficits and apoptosis by activating autophagy (Liu et al., 2017).

In addition to the alterations observed in PINK1 levels, our study also detected elevated levels of Asy-no in the plasma of both PD and PDs patients compared to healthy controls, consistent with several previous findings (El-Agnaf et al., 2006; Wang et al., 2015). This suggests that Asy-no could serve as a potential biomarker for PD diagnosis. However, it is noteworthy that the elevation of Asy-no was observed primarily in the modeling cohort and not consistently replicated in the validation cohort, potentially due to differences in sample characteristics or methodology. Interestingly, different α-synuclein forms displayed mixed results (Atik et al., 2016). One possible explanation for these conflicting results could be the variations in the methods used to detect the low concentrations of a-synuclein in biofluids. Currently, most biofluid biomarkers, including cerebrospinal fluid (CSF), urine, and plasma biomarkers, are primarily analyzed using ELISA techniques (Heikenfeld et al., 2019). However, these assays are often performed manually and can be challenging to standardize. Expanding the range of the testing methods and implementing more rigorous sample preparation protocols could enhance the precision of biomarker detection. Moreover, our study did not find any association of the PINK1 level with clinical scales commonly used to assess the motor severity and non-motor symptoms in PD, showing that PINK1 is a disease-sensitive but not a symptoms-specific marker of the extent of neurodegeneration. Of note, lower PINK1 levels were observed in PD patients with comorbid depression and anxiety in the PDs group. Due to the limited sample size and disproportionate distribution between the two groups in PDs, these observations should be carefully explained and require further validation in larger cohorts.

It is important to acknowledge the limitations of our study. First, the number of PD patients in the validated model was relatively small. Additionally, the lack of separate PD patients into “on” and “off” stages (only done in the “on” state of the disease) when evaluating neuropsychological tests, limited our investigation of the correlation of biofluid markers in plasma with the disease motor and non-motor severity of PD. Second, the cross-sectional study design cannot reach a causal relationship between plasma biomarker changes and the disease process in PD. Therefore, further longitudinal studies with serial measurements of plasma markers are warranted to address these limitations. Third, in this study, the diagnosis of idiopathic PD was established based on the widely accepted MDS clinical diagnostic criteria. Therefore, the potential influence of PINK1 mutations on our findings remains uncertain and warrants further investigation. Lastly, it is important to note that the diagnosis of PD in our cohort was based on clinical diagnostic criteria from the Movement Disorder Society (MDS) and lacked confirmation through neuropathological examination.

## Conclusion

We performed an ELISA-based analysis on plasma protein targets from a cohort comprising patients with PD and carefully matched control subjects to identify deregulated PINK1 levels. In summary, our findings suggest that elevated PINK1 in plasma holds the potential to serve as a non-invasive tool for distinguishing PD patients from controls. Longitudinal studies undertaken over a more extended period will be required to determine whether PINK1 can act as a potential biomarker of disease diagnosis and progression in the future.

## Data availability statement

The original contributions presented in the study are included in the article/Supplementary material; further inquiries can be directed to the corresponding authors.

## Ethics statement

The studies involving humans were approved by the Institutional Ethics Board Committee of the Wenzhou Medical University First Affiliated Hospital. The studies were conducted in accordance with the local legislation and institutional requirements. The participants provided their written informed consent to participate in this study.

## Author contributions

XH: Conceptualization, Writing – review & editing, Investigation. YZ: Writing – review & editing, Data curation, Investigation, Visualization. JH: Investigation, Writing – review & editing. TJ: Investigation, Writing – review & editing. YL: Data curation, Investigation, Writing – review & editing. SZ: Data curation, Investigation, Writing – review & editing. QY: Data curation, Investigation, Writing – review & editing. CX: Supervision, Writing – original draft. JL: Supervision, Writing – review & editing. WW: Supervision, Writing – original draft.
