# Supplementary material for: Detection of elevated levels of PINK1 in plasma from patients with idiopathic Parkinson’s disease
Source: Front Aging Neurosci. 2024 Apr 22;16:1369014. doi: 10.3389/fnagi.2024.1369014 (PMC11070528; doi:10.3389/fnagi.2024.1369014)
Supplement: Supplementary file 1 [file Data_Sheet_1.PDF]

Supplementary documents

Supple Table 1: PINK1 and ASO levels and clinical characteristics scores of participants were analyzed in univariable and multivariate logistic regression in cohort 1

Variables	Univariable analysis				Multivariable analysis			
	β	Wald χ 2	p value	OR(95%CI)	β	Wald χ 2	p value	OR(95%CI)
Age	0.032	4.402	0.036	1.033 (1.002, 1.064)	0.042	2.686	0.101	1.043 (0.992, 1.097)
Sex	-0.467	3.113	0.078	0.627 (0.373, 1.053)	-1.068	3.604	0.058	0.344 (0.114, 1.035)
Height	-0.015	0.74	0.39	0.986 (0.954, 1.019)				
Weight	-0.018	1.933	0.164	0.982 (0.957, 1.007)				
BMI	-0.042	0.983	0.321	0.959 (0.884, 1.041)				
Education	-0.02	0.313	0.576	0.98 (0.915, 1.051)				
Smoker	-0.488	1.908	0.167	0.614 (0.307, 1.227)				
Dringker	-0.405	1.52	0.218	0.667 (0.35, 1.27)				
HP	0.511	3.593	0.058	1.667 (0.983, 2.826)	1.107	6.078	0.014	3.027 (1.255, 7.301)
DM	0.063	0.034	0.854	1.065 (0.543, 2.089)				
MMSE	-0.058	5.453	0.02	0.944 (0.899, 0.991)	-0.003	0.005	0.945	0.997 (0.903, 1.099)
HAMD	0.208	23.015	<0.001	1.231 (1.131, 1.34)	0.178	3.649	0.056	1.194 (0.995, 1.433)
HAMA	0.15	24.064	<0.001	1.161 (1.094, 1.233)	0.023	0.126	0.723	1.023 (0.901, 1.162)
RBDQ-HK	0.059	23.469	<0.001	1.061 (1.036, 1.087)	0.04	5.713	0.017	1.041 (1.007, 1.076)
PINK1	0.057	40.914	<0.001	1.059 (1.041, 1.078)	0.048	14.078	<0.001	1.049 (1.023, 1.075)
Asy-no	0.002	39.215	<0.001	1.002 (1.001, 1.002)	0.001	13.792	<0.001	1.001 (1.001, 1.002)

All characters were assessed with univariate analysis followed by a multivariable analysis using logistic regression analysis between PD and HC. The characters showed significant between-group differences ($p < 0.1$) were included in the multivariable analysis.

Supplementary Table 2. Comparisons of the accuracy for combined models in distinguishing PD and HC in cohort 1

Model type	Variables in model	OR [95% CI]	AUC [95% CI]	*p*-value	AIC
**1. PINK1 + Asyno**	PINK1	1.042 (1.022, 1.065)	0.820 (0.761, 0.879)	0.975 vs 2	226.74
	Asyno	1.001 (1.001, 1.002)		0.522 vs 3	
				0.007 vs 4	
**2. PINK1 + RBDQ-HK**	PINK1	1.062 (1.043, 1.083)	0.829 (0.763, 0.875)	0.975 vs 1	246.94
	RBDQ-HK	1.065 (1.04, 1.095)		0.576 vs 3	
				0.013 vs 4	
**3. Asyno + RBDQ-HK**	Asyno	1.002 (1.001, 1.002)	0.833 (0.777, 0.89)	0.522 vs 1	221.84
	RBDQ-HK	1.059 (1.032, 1.091)		0.576 vs 2	
				0.028 vs 4	
**4.PINK1 + Asyno + RBDQ-HK**	PINK1	1.045 (1.023, 1.069)	0.861 (0.808, 0.913)	0.007 vs 1	206.8

	Asyno	1.001 (1.001, 1.002)	0.013 vs 2
	RBDQ-HK	1.059 (1.032, 1.092)	0.028 vs 3

Results are from logistic regression models using PD vs HC as the outcome in cohort1. *P*-values are from the comparison of AUCs (DeLong statistics). Abbreviations: AIC, Akaike information criterion (lower equals a better model fit); AUC, area under the receiver operating characteristic curve; CI, confidence interval; OR, odds ratio. Other abbreviations as in Table 1.

Supplementary Table 3: PINK1 levels from 3 diagnostic groups in different subgroups

Subgroup	HC		PD		PDS	
	Num	PINK1 level Mean ± SD	Num	PINK1 level Mean ± SD	Num	PINK1 level Mean ± SD
**Female**	62	73.562±14.406	91	95.208±19.926	21	92.795±21.526
**Male**	45	77.56±15.744	106	91.055±20.174	29	90.596±20.589
**Non-Obesity**	51	73.145±14.032	104	93.582±20.214	29	93.124±20.912
**Obesity**	56	77.154±15.788	93	92.293±20.093	21	89.302±20.944
**Non-HP**	52	74.584±15.512	135	92.306±19.082	24	97.587±21.391
**HP**	55	75.867±14.699	62	94.426±22.298	26	85.918±18.944
**Non-DM**	91	73.558±14.496**	164	92.773±19.677	37	90.935±21.667
**DM**	16	84.828±14.927	33	93.971±22.472	13	93.182±18.812
**Non-Smoker**	93	75.257±14.444	156	93.72±20.19	36	92.223±20.25
**Smoker**	14	75.154±19.196	41	90.134±19.819	14	89.71±22.837
**Non-Drinker**	89	74.085±14.012	150	94.83±19.747*	36	93.378±19.191
**Drinker**	18	80.972±18.778	47	87.048±20.347	14	86.74±24.59
**Non-Anxiety**	75	76.383±14.922	78	95.884±21.128	22	101.03±21.014**
**Anxiety**	32	72.573±15.219	119	91.065±19.276	28	84.05±17.609
**Non-Depression**	93	76.135±14.999	116	93.712±20.49	27	96.979±22.029*
**Depression**	14	69.319±14.469	81	91.915±19.645	23	85.11±17.618
**Non-CI**	72	74.941±14.61	115	93.685±21.334	10	87.18±14.004
**CI**	35	75.864±16.094	82	91.975±18.353	40	92.604±22.185
**Non-RBD**	91	74.332±14.991	108	94.573±19.907	31	95.072±22.403
**RBD**	16	80.428±14.725	89	91.032±20.309	19	85.722±16.847

All subjects with different diagnosis were divided into 10 subgroups and PINK1 levels were compared by t test between positive subjects and negative subjects in each subgroup. Significant values were added with "*" in the PINK1 level of each positive subgroup. * represents $p < 0.05$, ** represents $p < 0.01$. Obesity, subjects with BMI ⩾ 24; HP, subjects with high blood pressure; DM subjects with diabetes mellitus; Smoker, subjects who have smoked continuously or cumulatively for 6 months or more in their lifetime; Drinker, subjects who have consumed alcohol continuously or cumulatively for 6 months or more in their lifetime (at least once a week); Anxiety, subjects with possible anxiety and HAMA score ⩾ 7; Depression, subjects with possible depression and HAMD score ⩾ 7; CI, subjects with cognitive impairment according to MMSE scores (MMSE was used for cognitive examination, with adjustment of the cutoff score for cognitive impairment according to the years of education as follows: illiterate, ⩽17 points; primary school education, ⩽20 points; and postsecondary education or above, ⩽24 points); RBD, subjects with RBDQ-HK >18 points.
